# Children’s perceptions of natural history museums: Insights from the “Young Naturalists” program in Portugal

**DOI:** 10.1371/journal.pone.0357466

**Published:** 2026-09-18

**Authors:** José Luís Pimenta, João Carlos Paiva, Maria João Fonseca

**Affiliations:** 1 Faculty of Sciences the University of Porto, Rua do Campo Alegre, Porto, Portugal; 2 CIQUP, Unidade de Ensino das Ciências, Departamento de Química e Bioquímica, Faculty of Sciences the University of Porto, Rua do Campo Alegre, Porto, Portugal; 3 Natural History and Science Museum of the University of Porto (MHNC-UP), Praça Gomes Teixeira, Porto, Portugal; Instituto Federal de Educacao Ciencia e Tecnologia Goiano - Campus Urutai, BRAZIL

## Abstract

Children and adolescents are a key audience for scientific and cultural outreach and literacy. As future citizens, voters, and taxpayers, their education—carried out in formal, informal, and non-formal contexts—is crucial for societal development. In this context, scientific culture is decisive, and museums offer a particularly enriching environment. However, there is limited literature addressing young people’s ability to define the purpose of museums as infrastructures. Using an educational program developed at the Natural History and Science Museum of the University of Porto (Portugal), the “Young Naturalists” program, a study was developed aiming to fill in this gap. “Young Naturalists” engages participants aged 5–15 in thematic workshops, guided visits, and hands-on activities that replicate the everyday work carried at a natural history museum. Sessions are led by specialized staff from fields ranging from archaeology to science communication. The program’s emphasis on authenticity and access to backstage spaces aims to empower participants to engage with museum spaces, collections, and practices, fostering a deeper understanding of the institution’s mission and core activities. A qualitative methodology was adopted, consisting of three focus groups with a total of 21 participants. The study explored young people’s perceptions of the functions of museums, their attitudes towards museums, and the program’s impact on these dimensions. The findings indicate that children and adolescents primarily perceive museums as spaces for learning and heritage preservation, placing less emphasis on exhibition and display. Museum visits are associated with enjoyment and discovery, and participants value opportunities for active involvement, as well as diversity and technology-enhanced forms of engagement. These results can inform the design of participatory and age-inclusive programs that strengthen young people’s sense of belonging to natural history museums.

## Introduction

This research was conducted in the context of an educational program organised by the Museum of Natural History and Science of the University of Porto (MHNC-UP). The program, entitled Young Naturalists (YN), allowed children and adolescents to participate in practical thematic activities that replicate the work carried out by the museums’ curators and in-depth visits to the space. These were complemented by lectures on the various themes around which the university museum's collections revolve (palaeontology, anthropology, zoology, etc.).

The development of the scientific communication industry in Portugal is essential, given the country's position as one of the poorest and most underdeveloped in Western Europe **[1]**. Some authors have established a link between scientific literacy and communication and technological development, as well as the proper use of science and technology and an adequate understanding of the challenges humanity will face in the future **[2]**. Encouraging an interest in visiting museums (not only, but especially among young people) is an important mission.

Considering there is a lack of literature addressing children and young people’s ability to define the functions and the purpose of museums as infrastructures – the collection, preservation, and display of cultural heritage for the purpose of educating visitors – the YN program provides an opportunity for such. The study will also examine participants’ feelings toward the MHNC-UP and museums in general – that is, whether they consider visiting them to be a fun and enjoyable experience. Finally, it will examine the impact the program had on the two aspects mentioned earlier: whether it contributed to a better understanding of the roles of museums as institutions and whether it influenced participants’ appreciation of them.

### Museums as informal and non-formal learning settings

As informal education institutions, science museums aim to combine education with entertainment. Museums, regardless of the subject they address, are intended to evoke feelings of curiosity and fascination among visitors by displaying unusual, valuable, beautiful, or majestic objects. In the case of educational museums, such objects serve to illustrate scientific concepts, natural phenomena, and historical narratives **[3–7]**. Over the past decades, the museum visiting experience has been increasingly enhanced through multimedia experiences, interactive modules, and hands-on activities **[5,8–12]**. Museums also serve as platforms for social interaction – both among visitors and between visitors and museum educators **[3,13–15]**. Workshops, which combine hands-on activities and social interaction, have become increasingly prominent within museums’ educational offerings **[5,15]**.

Current scientific literature highlights that the various factors mentioned above – namely the diversification of sensory stimuli (including hands-on and physical activities), storytelling, and social interaction – contribute to the consolidation of learning. The emotional component of learning becomes evident: people learn best when emotionally engaged **[16–20]**. Such data is consistent with constructivist and social constructivist theories, which emphasize how learning is molded by the learner's broader context (rather than viewing learning as a passive process of absorbing knowledge) **[3,21–23]**. Falk & Dierking's Contextual Model of Learning (CML) posits that visitors’ experience and learning in museums are shaped by the interplay between the personal, the socio-cultural and the physical contexts **[3]**.

Children and adolescents represent a vital audience for science communication infrastructures such as museums. As future citizens, taxpayers and decision-makers, their early engagement with cultural and scientific institutions can influence their lifelong learning trajectories, civic participation, and relationship with natural and historical heritage **[5,15,18,24]**. Piscitelli and Anderson **[25]** found that tactile and narrative-rich exhibits significantly enhance recall and conceptual understanding among children aged 4–7. Munley’s **[23]** comprehensive review concluded that museums uniquely support early learning by fostering “islands of expertise” – a concept formulated by Crowley and Jacobs **[24]** – topics about which children develop deep knowledge through repeated exposure and social interaction.

The study on which this article is based did not assess the learning outcomes of participants in the YN project. It is clear, however, that facilitating learning in a museum setting—for young people and others alike—requires enhancing the visitor experience. And enhancing the visitor experience requires understanding visitors’ perceptions of museums.

### Children’s and teenager’s perceptions of museums

Research focusing on how children and adolescents understand museum functions remains limited. Much existing work addresses learning outcomes or visitor satisfaction. Across international contexts, researchers have explored children’s and adolescents’ attitudes toward museums, coming with mixed findings.

Children often describe museums as enjoyable places to visit, perceiving them primarily as places to learn, discover, and experience wonder. Feelings of boredom are not unheard of, though **[25–28]**.

In a study performed in Argentina involving over 130 children aged 8–13, Melgar **[27]** found that most participants were capable of identifying the purposes of *heritage preservation and exhibition* and *public education* when asked what a museum is: 56% of respondents saw museums as places «where things and ancient objects that are important to history or the past are shown, exhibited and kept», and other 35% saw them primarily as places «to learn, observe, get informed and get to know». Learning, curiosity, and fun were dominant associations, while boredom was rare.

Similarly, Hod-Shemer and Sher **[28]** observed that Israeli kindergarteners aged 5–6 described museums as large, fun places where «you learn new things». One in five children recognized museums’ role in preserving objects that are valuable from a scientific, cultural or historical point of view. Jensen’s **[26]** interviews with British children aged 9–10 revealed them to consistently perceive museums as places of learning but to have «mildly positive feelings» toward them, dividing their characterization of museums as «fun, boring, or a combination of the two» **[26]**.

Such perceptions are shaped by factors that include socialization patterns, technological expectations, and the degree of agency afforded to young visitors. Programs that emphasize co-creation, digital interactivity, and dialogue with professionals appear to enhance engagement and motivation **[5,8,9,11,12]**.

In contrast, research involving teenagers often portrays a more ambivalent or negative relationship with museums. Several European studies report that adolescents tend to perceive museums as boring, unwelcoming, and disconnected from their interests **[29–33]**. Using questionnaires with Spanish teenagers aged 14–16, Santacana Mestre et al. **[32]** found that many preferred activities such as sports or digital media over museum visits, citing a lack of dynamism and relevance.

Miras **[33]** similarly documented young people’s sense of exclusion, arguing that museums rarely consider adolescents’ voices when designing educational activities. Participants called for more interactive, visually stimulating, and technologically integrated experiences. Studies in Portugal have reached comparable conclusions: Cesário et al. **[8,9,29]** characterized teenagers as “experience seekers” who value social interaction, multimedia engagement, and gamified learning. Follow-up research using co-design sessions, Cesário et al. **[29]** found that adolescents envision ideal museum experiences as combining gaming, social connection, and hands-on exploration. Museums can also foster belonging and self-efficacy when young visitors see themselves reflected in narratives and collections, by addressing topics that interest them **[32,33]**. As Jensen **[31]** suggested, adolescents are highly sensitive to condescension but can be thoughtful and reflective when genuinely invited into dialogue.

## Methodology

### Context

In Portugal, the educational potential of natural history and science museums and science centers has gained increasing recognition over the last two decades, supported by the expansion of the network of science centers of the *Ciência Viva* agency. *Ciência Viva* was created in 1996 by the Portuguese Ministry of Science and Technology to promote scientific literacy **[34]**.

The Natural History and Science Museum of the University of Porto (MHNC-UP) stands out as a prominent player in the field of science communication. The Museum of Natural History and Science of the University of Porto (MHNC-UP) was formally created in 2015, resulting from the merger of the former Museum of Natural History and the Museum of Science, both of the University of Porto. There are two poles: the Core Pole, located in the building that houses the Rectory of the University of Porto and which preserves the bulk of the museum’s historical collection, and another pole, where the current Faculty of Sciences is located and where one can find the joint facilities of the Botanical Garden and the Hall of Biodiversity (which is part of the *Ciência Viva* network of science centers) **[35–37]**.

In 2023 the Museum launched *Pequenos Naturalistas* (*Young Naturalists*), an extended educational program designed to bring children and adolescents into sustained contact with the museum’s collections, researchers, and spaces. The program’s overarching goal was to foster curiosity and appreciation for the natural world while familiarizing participants with museum practices of collection, preservation, and interpretation. The program’s interdisciplinary nature made it an ideal setting to investigate how young participants construct meanings around the concept of museum.

The Young Naturalists program offers an opportunity to fill the gap in literature concerning young people’s notion of the nature and purpose of museums through a case study of how participation in a year-long program at a natural history museum influenced children and adolescents’ understanding of museums. Program participants are able to share their perceptions, together with their emotional appreciation of the museum, as well as the impact that their participation in the program had on both aspects. So, the research questions for this study are:

**RQ1:** Do children and teenagers understand what natural history museums and their collections are and serve for?

**RQ2:** What do children and teenagers think about natural history museums, and do they enjoy them?

**RQ3:** Did the YN project contribute to the participants’ factual understanding and valuative appreciation of museums? This study constitutes a contributes to a growing international discourse on how museums can cultivate sustainable relationships with young audiences.

### Research design and approach

The following case study adopted a qualitative, exploratory and interpretative approach. The analytical framework was informed by constructivist and social constructivist perspectives on learning and visitors’ experiences in museums. Children and adolescents are held as active participants in the construction of knowledge and meaning. In order to encourage interaction and discussion among participants, focus groups were deemed the most adequate method to elicit their thoughts and feelings **[38–42]**.

### Participants

Participants were randomly recruited from among the 45 children and adolescents enrolled in *Young Naturalists*. *YN* takes children and adolescents aged 5–15 to participate in thematic workshops related to the various scientific areas represented in the museum’s holding – zoology, botany, mineralogy, paleontology, archaeology, ethnography and history of science –, as well as to the efforts of collection, preservation, interpretation, and divulgation performed in the museum through hands-on activities, preceded by theoretical lectures and sometimes complemented by guided visits through the museum’s spaces. Participants are split into three groups according to their age range: one group for children between 5 and 7 years old (Group A, n = 15), another group for children between 8 and 11 years old (Group B, n = 16), and a last group for youth between 12 and 15 years old (Group C, n = 14). Participants represented a variety of socio-economic backgrounds, though most came from urban areas in and around Porto. Given the wide age range of the participants, comparisons were conducted across age groups, as defined in the YN program, in order to identify both cross-cutting patterns and age-related differences.

### Data collection and analysis

The recruitment for focus group participants took place in a period of ten days during 2024. Three focus group sessions were held in the month following the recruitment period. Semi-structured interview guides encouraged participants to discuss what they thought a museum was, what its purposes were, and how they felt during visits and activities.

No session exceeded 30 minutes in duration. Conversations were conducted in Portuguese and recorded. They were subsequently transcribed verbatim, translated into English by a linguistics professional from the Faculty of Arts and Humanities of the University of Porto and subjected to content analysis. Such content analysis was conducted by a single coder following Amado **[43]** and Bardin’s **[44]** guidelines. The coding system developed was validated by two experts directly involved in the study. First, transcripts were read repeatedly and in full to ensure familiarization with the dataset and to identify relevant units of meaning. They were then submitted to inductive (open) coding to identify patterns in the participants’ responses that could give rise to analytical categories. For example, in the analysis of participants’ definitions of museums and their perceived functions, responses were grouped into three main categories: *heritage display*, *heritage preservation* and *public education*.

Table 1 shows the script with the questions asked to the project participants in focus groups. The script was validated by experts prior to the focus group. It was not piloted due to time constraints imposed by the study's schedule. Not all topics covered in the questions will be discussed in this article.

**Table 1:** Script used for interviews.

**1.** What is a museum? **1.1.**What is the purpose of museums? **2.** Do you like visiting museums? **3.** Do you think you learn anything when you visit museums? **3.1.**A lot or a little? **4.** How do you think the objects in museums end up there? **4.1.**What precautions do you think need to be taken? **4.2.**Why is it important to keep and preserve these objects? **5.** Do you think that museums are available to everyone equally: rich and poor, people from different social classes, etc. **6.** Do you like visiting museums more now that you're Young Naturalists? **7.** Do you feel that you’ve learned anything with these activities? **7.1.**A lot or a little? **7.2.**What have you learned? (i.e., Can you give examples of things you’ve learned?) **8.** Which of the fields covered is your favorite? **9.** What did you like the most? **10.** What did you dislike or enjoy the least? **11.** What would you like to do here that you haven’t done yet? **12.** Would you take part in this project again? **13.** Would you visit the Central Pole or the Hall of Biodiversity again (i.e., outside the project)? **14.** Do you think museums are appropriate for your age?

### Ethical considerations

No personal data from any of the participants was collected. An Informed Consent Statement was drafted for the participants’ guardians, the terms of which were validated by the University of Porto's data protection officer. The guardians were informed in advance of the intention to conduct it and gave their informed consent in writing or by email. The children and young people participated in the study of their own free will. The recordings of the sessions were destroyed after transcription, ensuring anonymity. In the transcripts names are replaced by codes: thus, participants in Group A range from A1 to A8, in Group B from B1 to B7, and in Group C from C1 to C6.

## Results

The complete transcripts of the focus groups can be found in the Supporting Information. Analysis of field notes and focus group transcripts revealed four interrelated themes that describe how the children and adolescents who took part in the study perceive and experience natural history museums:

- Understanding the nature, purpose and value of museums;
- Emotional and affective engagement with museums;
- Learning and participation in museum contexts;
- Impact of the *Young Naturalists* program on perceptions and attitudes regarding the aforementioned topics.

### Understanding nature, purpose and value of museums

Overall, 15 participants answered the questions «*What is a museum?*» and «*What is a museum for?»*. Three fundamental aspects could be identified in their responses: (1) *exhibition of heritage*; (2) *preservation of heritage*; and (3) *public education*.

In the context of this topic, a total of 27 text segments were coded:

- 7 referred to *exhibition of heritage* (2 for Group A, 3 for Group B and 2 for Group C);
- 10 to *preservation of heritage* (2 for Group A, 2 for Group B and 6 for Group C); and
- 10 to *public education* (1 for Group A, 7 for Group B and 2 for Group C).

*Preservation of heritage* and *public education* are on equal footing in terms of mentioning frequency, with the first being particularly emphasized by Group C (6 out of the group’s 10 codes) and the second by Group B (7 out of 12).

*Exhibition of heritage* was the least common, receiving particular attention in Group A (2 codes out of 4) where it matched *preservation of heritage* and outperformed *public education*. In Group B it outperformed *preservation of heritage* but was largely supplanted *by public education*, whereas in Group C it matched *public education* but was largely supplanted by *preservation of heritage*.

**Fig 1:**
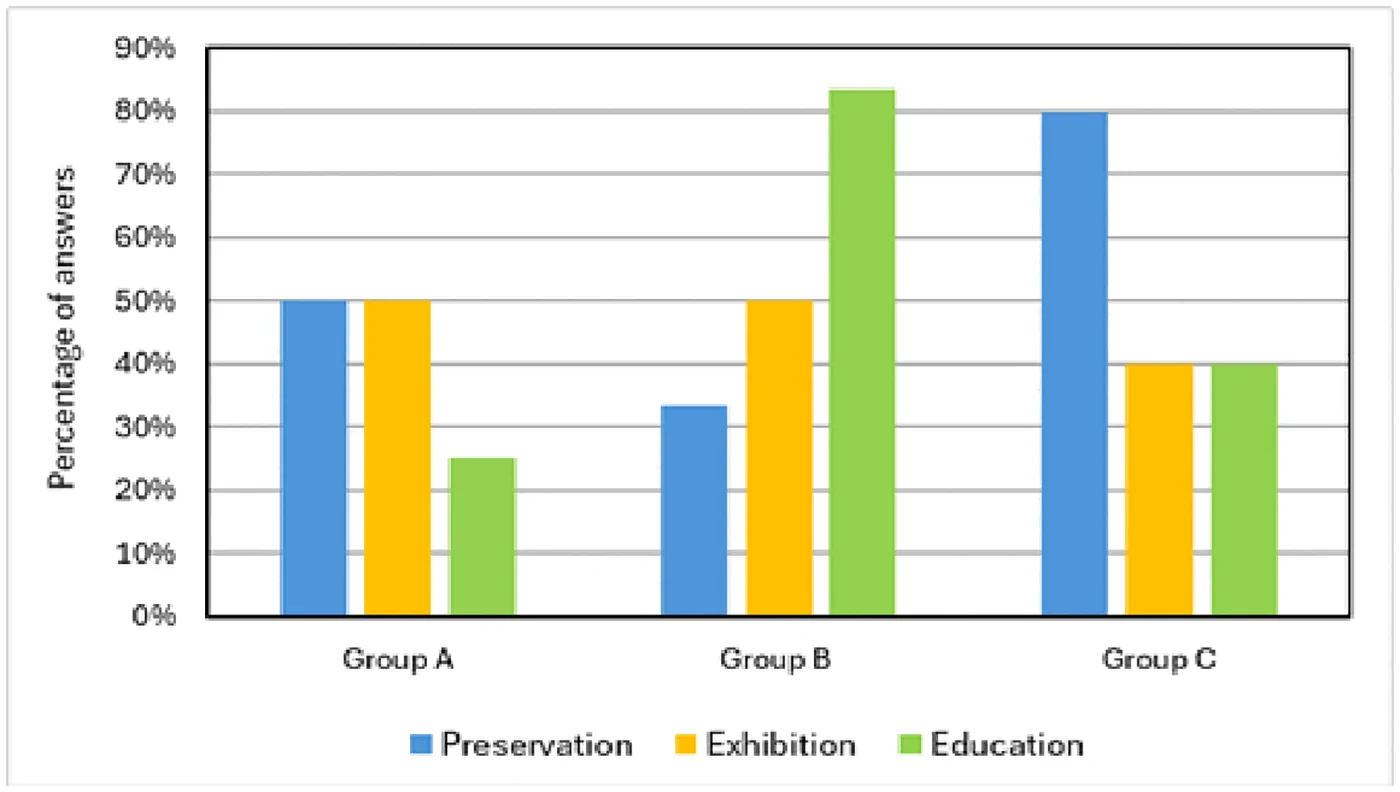
Percentage of answers to questions «*What is a museum?*» and «*What is a museum for?*» by purpose. Purposes are separated preservation of heritage; exhibition of heritage and public education for each group: A, B and C.

By quantifying the number of participants who refer to each code and calculating their percentages in the context of each group (Fig 1), we arrive to the following results (n = 15):

- 7 (47%) addressed the topic of *exhibition of heritage* (2 for Group A, 3 for Group B and 2 for Group C),
- 8 (53%) addressed the topic of *preservation of heritage* (2 for Group A, 2 for Group B and 4 for Group C) and
- 8 (53%) addressed the topic of *public education* (1 for Group A, 5 for Group B and 2 for Group C).

It should be noted that the three categories are not mutually exclusive: a participant may mention more than one aspect in their response.

Apart from a slight drop in the percentage of participants who point to the exhibition of heritage aspect with age (50% for Groups A and B and 40% for Group C), no age-related trend can be extrapolated. Both the quantification of codes and the quantification of participants who pointed to a certain aspect show that the children and adolescents viewed exhibition of heritage as secondary in relation to the preservation of heritage and public education.

Question «*Why is it important to keep and preserve these objects* [the objects kept in museums]?» also refers to the functions assigned to museums. This question was not put to Group A, which therefore did not comment on the matter. Among the answers given by Groups B and C, two fundamental aspects could be identified: (1) *contemplation* and (2) *education*. In the context of this topic, a total of 12 text segments were coded:

- 2 referring to *contemplation* (one for each group)
- 10 referring to *education* (6 for Group B and 4 for Group C)

All participants agreed that museums are spaces that promote learning on a large scale. None of them had criticism to direct to the way museums provide and organize information.

These results resonate with previous findings by Melgar **[27]**, who reported that children primarily define museums as spaces for learning about the past. They also align with Hod-Shemer and Sher’s **[28]** observation that even very young children associate museums with knowledge and care, though explicit notions of conservation increase with age.

### Emotional and affective engagement with museums

In Groups A and C, participants unanimously and immediately declared they enjoyed visiting museums (Tables 2 and 3).

**Table 2:** Excerpt from the focus group with Group A.

**JP** **(Researcher):** Now, one by one: do you like visiting museums?/ All: [In unison.] Yes!/ **JP**: We didn’t even need to hear you one by one because you’ve all said the same thing!

**Table 3:** Excerpt from the focus group with Group C.

**JP**: Do you like visiting museums?/ All: [Unison, quietly.] Yes./ **JP**: Everyone says yes, right?/ C4: Yes…

As shown in Table 4, participants in Group B also reached the consensus that visiting museums was generally a pleasant experience, though that was preceded by a debate in which participants discussed how factors such as the kind of museums one visits, and the topics addressed in said museums influence one's appreciation. Diversity of topics and objects on display was highlighted as a factor contributing to the attractiveness of the experience.

**Table 4:** Excerpt from the focus group with Group C.

**JP**: There you go! Do you like visiting museums?/ All but B4 and B5: [In unison.] Yes!/ B4: It depends!/ B5: It depends!/ B4: Yeah! [Dismissive tone] If it is one of those, like… the body./ B7: If it is, like, animal museums… It depends on what I see. I prefer museums that have more diversity, that don’t show the same stuff all the time. […] B5: A variety of things!/ B4: It depends. Some museums are kind of boring. Like, they have…/ B7 and B5: The same things all the time! […] **JP**: Nice! But then, deep down, do you think you all like visiting museums?/ All: [In unison.] Yes!

To what extent do young people feel «at home» in the museum is one of the central questions of this study. Group A unanimously declared that museums are «*for everyone*».

In Group B, this topic was spontaneously addressed beforehand by one of the participants in the context of question «*Do you think that museums are available to everyone equally: rich and poor, people from different social classes, etc.?*» (Table 5). This question addresses the importance of social conditions in people’s access to museums, but the participants led the discussion to the people's access according to age.

**Table 5:** Excerpt from the focus group with Group B.

B1: Museums are more for grown-ups, generally…/ **JP**: We’ll get there! We’re going to talk about that too!/ B5: I don’t think so… I think it’s, like, for all ages./ B1: [Replying to B5.] Nobody would bring a baby! He can’t just be there going «naaaaah» [imitating a baby crying] …

When the question was put to them directly (Table 6), B1 adjusted their position, arguing that a museum’s suitability for children depends on the disciplines it covers and how these are presented. B6 and B4 agreed, adding that themes such as the human body tend to be more oriented toward adults. B7 disagreed, insisting that the variety of subjects and objects found in museums makes them attractive to all ages. Later, when the question was reframed «*Do you think museums take people your age into account, or are they designed only for adults?*», none of the participants supported the idea that museums are created exclusively for adults.

**Table 6:** Excerpt from the focus group with Group B.

**JP**: Right! Now, this has to do with something that was said earlier: do you think museums are appropriate for your age or do you think they're for older people?/ B1: It depends!/ B6: It depends on the subject of the museum!/ **B1:** Sometimes it seems to be more for adults, other times…/ **B4:** Like, if it's the human body, like, the heart and stuff, I think it's more for adults…/ B6: The veins! […] B7: I think it's for all ages for one reason and one reason only: because museums don't have just one thing, they have many things!/ **JP**: But do you think that the way museums are organized […] takes into account people your age or do they only think about adults?/ B1: No!/ B7: No! They have…/ B4: No!/ B6: I say: all ages!

As shown in Table 7, in Group C, subjects addressed, dynamism and visitors’ maturity and individual preferences were pointed to as factors to consider. Once again, the human body was considered a topic which could be “scary” or discomforting.

**Table 7:** Excerpt from the focus group with Group B.

**JP**: [..] Do you think museums are appropriate for your age? […] Or are they more for adults?/ C1: No!/ C4: No!/ C3: No! […] C4: There are museums that are more dynamic and there are others that are… well, not boring, but…/ C3: Some have things that can sometimes scare people./ **JP**: Scare people? Like what? Can you name something?/ C3: Uh… I think there was one time when my cousin, when he was younger, went on a field trip to a museum and he fainted… [Laughing shyly.] It was something about the human body he had seen. […]/ C5: That also depends on the person's knowledge and what they do. It doesn't have so much to do with age, it can have more to do with maturity, with tastes, for example, I can go and see a museum of… [thinking] African colonial history and I might find it interesting and 90% of people might find it uninteresting. […]/ C4: It also depends on people's tastes, not just their maturity. And interests.

Ultimately, however, participants expressed an interest in visiting museums. Among children, scientific literature does not report extensive feelings of aversion to museums or of exclusion in the museum environment due to a putative “adult-centric” conception of museums **[25–28]**. Such feelings of fondness and integration, however, are strange coming from the older participants in light of the paradigm that emerges from the studies, according to which adolescents tend to feel out of place in museums and not to enjoy them very much **[30–33]**. The fact that the respondents are participants (most likely, *voluntary* participants) in an educational program carried out in a museum, which aims to familiarise them with and instil in them a taste for the space, may be behind their positive assessment. It is also worth noting the small number of respondents.

### Impact of the Young Naturalists program on perceptions and attitudes

All participants surveyed said they enjoyed the project, though some themes and activities caused boredom or discomfort.

A consensus was reached that the project made them appreciate visiting museums more and that they had learned a lot. In Groups B and C, comparisons were made with the school environment that were favorable to the YN project, highlighting in the latter the dynamism, liveliness and advantage of learning from experts. These results are in line with what the literature says about active participation, opportunities for interpersonal interaction and co-creation of content, and sensorially stimulating experiences. In addition to stimulating learning, these factors strengthen young people's enjoyment of visiting museums **[5–9,11,12,16–20,29,32,33,45–47]**. Several authors have called for collaboration between school education and non-formal means of education as a way of enhancing learning. Such a recommendation comes in the context of the call for more active and emotionally rewarding learning, which has been introduced more consistently and thoroughly in these non-formal means of education than in schools, and which differs from the traditional passive lecture-based teaching method **[5,48–50]**.

The participants’ positive assessment of the program may be attributed (at least partially) to the fact that it is based on a combination of pedagogical methods that the scientific literature considers particularly effective in engaging young people and promoting their learning: primarily hands-on practical activities, personal interaction (both among the youth themselves and between the youth and the facilitators), and the use of technology capable of providing audiovisual and interactive experiences [8,9,14,17,19,20,51,52**].**

The *YN* project aimed, among other things, to get participants to understand the function and importance of museums and to enjoy visiting them more. Participants universally declared that participating in the project had made them appreciate visiting museums more.

When asked whether they would return to the Museum outside the scope of the program, all the participants said yes.

At the affective level, participants’ enthusiasm for returning to the program or visiting similar institutions illustrates a strengthened emotional bond with museums. They viewed them as places not only for learning but also for leisure and friendship. Importantly, no participant expressed overtly negative or exclusionary sentiments about the museum’s accessibility or relevance – contrary to reports of alienation among teenagers in other studies **[30–33]**. This contrast may stem from the program’s emphasis on inclusion, informal communication, and consistent educator presence, which together fostered familiarity and trust.

Finally, participants’ suggestions for future editions of the program – new topics to address and new activities to perform – indicate an emerging sense of co-creation, co-design, engagement and agency, aligning with contemporary calls for youth-centered and co-created museum practices **[9,32,33]**.

## Conclusions

Taken together, these four themes reveal a coherent picture of how children and adolescents conceptualize and experience natural history museums. Their perspectives combine cognitive understanding, emotional connection, and social interaction. Education and preservation dominate their factual definitions, while enjoyment, curiosity, and belonging shape their affective evaluations. Learning is experienced as embodied and collaborative, and sustained participation strengthens appreciation and agency. This is consistent with constructivist and social constructivist approaches to museum learning **[3,21]**. Participants’ responses indicate that museum meanings were shaped through the articulation of prior knowledge, direct experience with collections, interactions with educators, curators and peers.

They also confirm the potential of natural history museums to serve as effective platforms for science communication and citizenship education, especially when programs are designed around participation, dialogue, and continuity.

### Implications for museum education practice

The findings of this study underscore the pivotal role that natural history museums can play in nurturing young people’s curiosity, learning, and cultural identity. They also reveal how children’s and teenagers’ conceptualizations of museums evolve through participatory engagement. For museum educators and program designers, these insights carry several practical implications.

### Design programs that integrate knowledge and emotion

The results of this study are consistent with the paradigm that emerges from scientific literature: children and adolescents perceive museums not simply as repositories of information but as spaces where learning is emotionally and socially charged **[3,5,6,8,9,24–34]**. Enjoyment, curiosity, and awe are powerful motivators for engagement, reinforcing the principle that cognitive and affective dimensions of learning are inseparable – in line with the social constructivist paradigm. Narratives, sensory stimuli, and storytelling can amplify emotional connection while supporting conceptual understanding. Museum educators should therefore design activities that intentionally weave together factual content and emotional resonance **[3–20,**^45^–^52^]. The three contexts present in Falk & Dierking’s CML are mapped onto the program’s specific design features: voluntary enrolment as a motivational filter (personal context), the richness of interactions between the children and between the children and the docents (socio-cultural context) and the majesty and richness of audiovisual stimuli of the MHNC-UP as backstage space **[3]**.

### Promote hands-on, inquiry-based participation

Across all age groups, participants highlighted the hands-on practical activities they had performed in their recalling of the project. Handling real objects, conducting small experiments, and engaging in creative tasks fostered learning and amusement. Museums can build on this preference by developing inquiry-based workshops that encourage children to explore questions, collect data, and share interpretations rather than merely receive explanations. Preparing herbarium samples, observing insects, or analyzing fossils are examples of activities young people can perform in museums as to embody the role of scientists. Similar models could be adapted for temporary exhibitions, weekend family sessions, or school partnerships **[5,51,52]**. Moreover, scaffolding – providing subtle guidance while allowing autonomy – proved essential. **[23]**.

### Balance digital innovation with authentic material experience

Although participants valued the use of technology in the activities they carried out, they highlighted hands-on activities and the contact with concrete objects in their recalling of the program. This observation reaffirms the importance of physical items and experiences in the face of increasing digitisation. While touch screens, gamification and virtual reality simulations can contribute to learning and enjoyment, they should not substitute reality **[12,53–55]**. One of the corollaries of the so-called “Cave Man Principle” (formulated by Japanese-American physicist Michio Kaku) is the human desire for physicality, for tangible, face-to-face experiences, rather than virtual alternatives **[54]**.

A balanced approach would integrate digital tools to support exploration—such as magnifying virtual models or accessing contextual information—while maintaining tactile and sensory dimensions. Technology can also facilitate post-visit engagement through online games or digital storytelling, reinforcing what has been learned on-site **[8–12,30]**.

### Encourage youth voice and co-design

The teenagers’ willingness to suggest improvements to the *YN* program demonstrates the feasibility and benefits of co-design. Involving young audiences in decision-making processes—from choosing topics to testing prototypes—can enhance the perceived relevance of museums and the motivation to engage with the museum environment. Co-creation also helps shift the museum’s image from an institution that speaks to young people to one that learns with them **[8,9,32,33,45]**.

### Build interdisciplinary and community partnerships

The success of the *YN* program was amplified by collaboration between educators, scientists, and communication professionals. Such interdisciplinary teamwork allowed participants to experience different perspectives—from scientific research to heritage interpretation. Expanding these partnerships to include schools, families, and local organizations can further enhance the program’s reach and sustainability. For example, aligning museum activities with school curricula can help teachers integrate informal learning outcomes into classroom practice. Engaging families through open days or collaborative projects can extend learning beyond the museum, reinforcing social support for science and heritage education. Partnerships with community groups also contribute to inclusivity, ensuring that programs reach diverse audiences and reduce barriers related to access or cultural capital **[5,24,50]**.

### Recognize the museum as a space of belonging

Participants’ feelings of comfort and inclusion highlight the museum’s potential as a space of belonging. Friendly communication, approachable educators, and opportunities for dialogue were essential in countering the stereotype of museums as formal or elitist institutions **[31,56–58]**. Museum staff training should therefore include strategies for cultivating relational warmth—active listening, humor, empathy, and validation of children’s ideas.

Furthermore, integrating social spaces within exhibitions—such as discussion corners, collaborative stations, or reflection walls—can encourage visitors to share their thoughts and experiences. These practices promote what Martinez **[57]** calls “shared authority,” where visitors’ voices contribute to meaning-making. In turn, such inclusivity can strengthen the social legitimacy of museums as participatory institutions serving diverse publics.

### Synthesis

This study examined how children and adolescents conceptualize natural history museums and how these perceptions evolve through participation in the *YN* program at the Natural History and Science Museum of the University of Porto. The research revealed that young participants hold rich, multifaceted understandings of museums as spaces of learning, preservation, and enjoyment. Their views integrate cognitive, emotional, and social dimensions, demonstrating that engagement with museums extends far beyond the acquisition of factual knowledge.

Three central insights emerged. First, participants viewed museums as trustworthy educational institutions that safeguard collective memory while fostering discovery. Second, affective experience – enjoyment, curiosity, and belonging – proved integral to learning, confirming that emotion is not ancillary but constitutive of museum education. Third, hands-on, inquiry-based participation enabled children and teenagers to construct meaning actively, reinforcing the value of experiential and collaborative learning.

These findings contribute to international museum-education scholarship by illustrating how longitudinal, participatory programs can nurture both knowledge and identity among young audiences. They also highlight the distinctive potential of natural history museums to bridge science and culture, combining authentic material encounters with opportunities for reflection and creativity.

In sum, the findings advocate for a model of museum education that is interactive, emotionally resonant, inclusive, and sustained. Natural history museums, with their unique blend of scientific and cultural heritage, are ideally positioned to implement such an approach. By integrating hands-on and inquiry-based activities, balancing digital and material engagement, fostering dialogue, and nurturing a sense of belonging, museums can cultivate the next generation of learners, citizens, and stewards of natural heritage. For educators and curators, the challenge is not only to communicate knowledge but also to create conditions in which young people feel seen, valued, and inspired to contribute to the shared stories that museums preserve.

### Limitations and future research

The study’s scope was limited to one institution and a relatively small sample, which constrains the generalizability of results. Likely, participants were already interested in the topics covered by the museum (hence their participation in the program), thus the collection of their perceptions may be biased and not representative of the young population as a whole.

Future studies could take advantage of projects similar to YN, carried out in other museums and science centres, by examining the longitudinal effects on participants’ perceptions, attitudes, and learning (an aspect that was not addressed in this study) through quantitative or mixed-method approaches. Comparative or multi-site designs might explore variations across museum types, cultural settings and socio-economic contexts.

Further research could investigate how different pedagogical models—such as digital co-creation, family learning, or outdoor exploration—shape young people’s emotional connections to museums.

## Supporting information

S1 File Focus Group A. Transcription of the focus group with Group A.
(PDF)

S2 File Focus Group B. Transcription of the focus group with Group B.
(PDF)

S3 File Focus Group C. Transcription of the focus group with Group C.
(PDF)
