# Supplementary material for: Children’s perceptions of natural history museums: Insights from the “Young Naturalists” program in Portugal
Source: PLoS One. 2026 Sep 18;21(9):e0357466. doi: 10.1371/journal.pone.0357466 (PMC13588495; doi:10.1371/journal.pone.0357466)
Supplement: Supplementary file 1 [file pone.0357466.s001.pdf]

FOCUS GROUP A

Players: JP, MF, CC, 12, 20, 32, 33, 41, 42, 43 and 52

JP Thank you all for being here so we can talk a little bit. I'm going to ask you a series of questions and I'd like you to talk to me, but also a bit amongst yourselves, okay? The first question is: what is a museum?

43 It's where people go to see things.

[*Silence.*]

JP [*Recording.*] It's where people go see things, exactly…

42 And ancient things.

12 It's where collections are stored!

33 It's where we go to learn about things from the past that we don't know… and want to learn about!

JP We already have here some very good answers! Does anyone else want to add anything? Do you want to comment on something one of your friends said?

[*Silence.*]

JP Nothing? Don't be shy! There's nothing to fear! Speak up! (Pause.) So… You said all this stuff about the museum, and you've somewhat already answered the next question, which is: what do you think is the point of a museum? It's more or less the same question, isn't it? I think we can move on… Now, one by one: do you like visiting museums?

All [*In unison.*] Yes!

JP We didn't even need to hear you one by one because you've all said the same thing! Next: you think you learn anything when you visit museums?

All [In unison.] Yes!

JP Same thing. How much do you think you learn? A lot? A little?

All [In unison.] A lot!

JP Now, how do you think the objects that you see in the museum, or in the museums, get there? The stuffed animals, the historical artifacts… What do you think happens for them to end up there?

43 People go searching for them and take them.

41 They go «exploring the objects».

JP [*Taking notes.*] Nice!

42 They go, they find them, and they bring to the museum!

12 Some archaeologists dig stuff up and then it can be taken to the museum.

JP Nice!

52 They find them and then dissect them!

JP That’s what happens to the animals, isn’t it? What precautions do you think need to be taken with what's in a museum?

52 You must be careful with the objects, which can be fragile.

41 Imagine they’re ancient…

JP [*Taking notes.*] Exactly!

10 Not getting it dirty! [*Repeating quietly.*] Not getting the things dirty…

41 Not touching… some of them.

52 That’s what I said! Only in a different way.

JP This next question might be a bit weird. Do you think museums… [*Pause, thinking.*] Consider this: do you think museums are available for everyone? Do you think everyone has the same ability to go and visit the museums or do you think it’s a bit unfair, because it is more for rich people, people who have a lot a money…?

All [*In unison.*] No!

43 Everyone has the right!

JP Yes, everyone has the right. But do you think… (*Pause, thinking.*) You know… Sometimes it is not even about rights; sometimes, there are people that can’t go because they don’t have a lot of money. But do you think that the way things are nowadays, I don’t know, like, in society, everyone can go to the museum equally, without any problems? Everyone, I mean, most people...

All [*In unison.*] Mostly, yes!

JP Now, do you think you enjoy visiting museums more now that you are Little Naturalists?

All [*In unison.*] Yes!

JP Do you feel like you've learned anything with these activities?

All [*In unison.*] Yes!

JP Do you think you've learned a lot? A little?

[*General nodding.*]

JP A lot, right? What did you learn? Can you tell me?

41 We've learned there are things which can be unexpected!

JP Nice!

52 That there are different kinds of plants! That are classified in different kinds of ways!

41 We've learned about nature and all that stuff!

JP Nice!

52 For me, I think it was more… like, the mammals.

JP You learned more about mammals? Nice! Now, from all the subjects we explored until now which were your favorite?

41 All of them!

JP [*Taking notes.*] All of them…

12 All of them!

43 All of them!

42 I didn't do a lot yet but… do I need to pick one? Mammals!

43 Wait, we've already explored mammals, how can you say you enjoyed them?

MF He/she likes them in general!

JP Let's ask one by one… (*To* **32**.) What did you enjoy the most?

32 Everything!

JP Everything?

32 Everything!

JP Nice! [*To* **43**.] What did *you* enjoy the most?

43 Everything!

41 I enjoyed everything as well!

JP [*To all the children.*] You enjoyed everything? Don't you have a favorite subject?

? No!

41 I enjoyed everything! Absolutely everything!

JP Nice, nice!

MF And was there a subject you thought you weren't going to enjoy that you ended up enjoying? Or did you already enjoy all these subjects before?

52 I thought I wasn't going to like the secret passage very much!

MF Which one?

52 The secret passage!

MF The secret passage… You mean archaeology, right?

42 What secret passage?

MF Archaeology! We built a cave!

[*Voice overlap.*]

42 When can we go there?

MF You'll have to wait until next year…

42 [*Disappointment.*] Oh…

JP Okay, now: what did you enjoy the most?

All [*In unison.*] Everything!

JP Yeah… I think you all agree there…

? [*Humming.*] Everything, everything, everything…

JP So, you’ve enjoyed everything. What did you enjoy the least?

43 Nothing!

12 Nothing!

(**MF** *laughs.*)

43 I did not dislike anything. We enjoyed it all!

JP Yes but think about it this way: you enjoy everything but still find some things more interesting. Was there anything you found less fun or was it all the same?

All [*In unison.*] It was all the same!

JP All the same? Absolutely?

? [*Singing.*] Everything, everything…

43 Everything was ok!

CC [*Joking.*] I though you enjoyed the plants more than anything!

(*Laughter.*)

MF [*Joking.*] No influencing!

JP Now, what would you like to do here that you haven't already done? Any ideas? Any suggestions?

41 Reptiles! I really like snakes!

12 New topics!

42 I know: we dissect an animal!

12 [*Repeating.*] New topics!

42 We dissect a fish!

JP [*To all.*] New topics?

12 [*Humming.*] New topics, new topics…

JP Someone over there [*at the end of the table*] said something I couldn’t hear!

42 Dissecting a fish!

12 Someone said catching a fish, right?

MF *Dissecting* a fish, yes.

42 *I* said dissecting a fish!

[*Voice overlap.*]

JP [*To* **20**.] What about you, **20**? Aren't you saying anything? You're very quiet!

(**20** *cringes and doesn't say anything.*)

43 He/she's shy…

41 He/she's shy…

? He/she doesn't say anything.

JP Would you take part in this project again if you had...?

All [*In unison, answering straight away.*] Yes!

JP Everyone, right? The same thing!

? [*Humming.*] Yes, yes, yes!

JP Would you visit the Biodiversity Gallery or the Central Pole again?

All but 42 [*In unison.*] Yes!

42 I haven't been there yet...

MF [*To* **42**.] Right, you haven't been to the Central Pole. That's another part of the museum.

[*Voice overlap.*]

MF [*To the others.*] Would you like to explain what you saw at the Central Pole?

42 No! I want it to be a surprise!

MF You want it to be a surprise? All right, then!

JP Okay! And now: do you think museums are appropriate for your age or do you think they're more for grown-ups?

All [*In unison.*] No!

? For our age too!

[*Voice overlap.*]

JP It’s for everyone, isn’t it?

41 So everyone can have fun and learn!

JP There you go! Okay, I think that's it... Thank you!
