# Supplementary material for: Children’s perceptions of natural history museums: Insights from the “Young Naturalists” program in Portugal
Source: PLoS One. 2026 Sep 18;21(9):e0357466. doi: 10.1371/journal.pone.0357466 (PMC13588495; doi:10.1371/journal.pone.0357466)
Supplement: Supplementary file 2 [file pone.0357466.s002.pdf]

FOCUS GROUP B

Players: JP, MF, 10, 11, 20, 30, 31, 50 and 51

JP I'm going to ask you some questions. You can answer them to me, but you can also talk a bit among yourselves, so there's some interaction. I'll record your answers. First of all, what is a museum?

10 It's a place where old objects are stored that may be important in the future.

31 Yeah, it’s basically what he said…

50 Right!

20 Yes…

JP Nice! [*Silence.*] Anything else you want to add?

11 I want to say something!

JP Go ahead!

11 I think museums, like 10 said, are places where you store old and rare objects which... [*Thinking.*] can be used... [*Thinking.*] in the future or they can just be there for display, so people can see them and learn more about them.

JP Nice! You added a lot of information! Does anyone have anything else to say?

11 No. They said it all…

JP Right! There isn’t much [to say] …

31 [*Interrupting.*] There isn’t much of a definition!

JP But you can always have something more to say!

11 [*Clarifying.*] Another detail…

[*Silence.*]

50 There's a new comma, whenever a tale is told![11]

11 What?! [*i.e., «What was the point of that?!»*]

---

[1] This is a Portuguese proverb: «*Quem conta um conto acrescenta mais um ponto.*» It refers to people’s tendency to recount a particular event differently, adding details of their own. Here it is a spontaneous *non sequitur*.

50 It's true!

JP [*Laughing, then moving on.*] What do you think a museum is for? You already said…

31 It's for… We said it already! I mean, it's for people to see objects from the past. That's it…

51 So they can observe and learn more…

31 Yes.

[*Voice overlap.*]

31 So they can have more knowledge…

11 Basically, it's for learning about ancient and rare things, which were explored by other people…

31 Learning more!

30 Yes! To learn!

20 Right! To learn! [*Laughs.*]

11 It's a summary of the thing we all said! Basically…

JP There you go! Do you like visiting museums?

All but 20 and 31 [*In unison.*] Yes!

20 It depends!

31 It depends!

20 Yeah! [*Dismissive tone*] If it is one of those, like… the *body*.

11 If it is, like, animal museums… It depends on what I see. I prefer museums that have more diversity, that don't show the same stuff all the time. Which, have things, like… [*Thinking.*]

31 A variety of things!

11 Exactly!

20 It depends. Some museums are kind of boring. Like, they have… [*Thinking.*]

11 and 31 The same things all the time!

20 Right! And there are others that, like …

10 [*Referring the boring museums.*] Like, always a vase…

[*Laughter.*]

20 But there are others that have cool stuff.

11 True!

31 Like 11 said, like, if I go to a museum that, like 10 said, only has vases… it's not fun.

[*Laughter.*]

31 But if there are, like, museums with animals, with objects…

50 *That* is more fun.

31 It's more fun, yes! It like it a bit more.

JP Nice! But then, deep down, do you think you all like visiting museums?

All [*In unison.*] Yes!

JP Yes! I guess one can say so! Do you think you learn anything when you visit museums?

All [*In unison.*] Yes!

50 A lot!

11 Like, where do things come from…

50 How they are…

11 How they were captured!

50 How they were created.

11 How things began!

20 How they started to exist…

11 [*To 20.*] How they started to exist, yes…

31 [*Joking.*] We're all so smart…

[*Laughter.*]

11 These questions aren't that hard…

JP Of course! This is not a test. And how do you think the objects in museums end up there? The embalmed animals, the historical artifacts…

10 They're treated before [*i.e., before being displayed*] by… [*Pause, thinking.*] well, I don't know what they're called!

31 They can also just be replicas.

11 Protections! [*i.e., protections are put*]

50 It's just replicas most of the time!

[*Voice overlap*.]

31 And it can be hard for someone to catch a whole dinosaur!

11 For example, that skeleton of a blue whale, that was still a teenager, I think that's a good example! A part is the actual skeleton, and another part is just replicas…

JP That's right! They couldn't preserve it all! So, they had to replace…

31 [*Interrupting.*] As I said: it's very difficult, or almost impossible to find a…

10 [*Completing.*] A complete skeleton!

31 Right!

JP It is not impossible, but it is highly improbable.

[*General agreement.*]

11 They [*i.e., the curators*] must be very good and take very good care of it.

JP What precautions do you think need to be taken?

11 A lot of them!

31 All the precautions in the world!

10 Embalming…

20 Cleaning…

50 You must be very careful, because if it breaks, it can… [*Pause, thinking.*] It’s gone!

10 And to put protections.

11 And, like, always having a replica of it.

31 Like, an ancient box, it is better to always have a replica of the box because it can break, you know…

[*Voice overlap.*]

50 And they must hide them all the time.

11 And usually, replicas have to be as similar [as possible] to the real thing, don't they? If they weren’t, anything would serve as a replica!

JP Right! And why do you think it is important to protect and preserve these objects?

31 So people can learn!

50 Exactly! So everybody can take a look. To know them later…

11 To know the past.

10 Like, when we are in the future, we can see how things really were a long time ago.

50 Exactly! And to know the world better!

31 Now [*i.e., modern age*] is the future of most things in the museum.

11 And not just for us to learn. To teach others one day, too...

JP There you go! [*Pause, recording.*] Don’t you want to say anything else about this?

50 Uh… No.

11 No.

JP It’s basically this, isn’t it?

31 Were’s so smart!

[*Laughter.*]

JP The following question might be a bit weird: do you think museums are equally available for everybody, or are they more for rich people, higher-class people.

50 I don't think so!

10 For everybody!

[*Voice overlap.*]

11 For everybody, but like… [*Pause, thinking.*]

JP I mean, we all have the right, but do you think our society is a bit unfair in the way it...

10 [*Interrupting.*] Yes.

50 [*Agreement.*] Hmm-hmm.

11 A lot! It values rich people more!

[*Voice overlap.*]

11 It's very unfair! Rich people are like: «Hand over all your money!». And then poor people are left in the corner…

JP But do you think that most of the museums you know or have visited take care to include everyone?

All [*In unison.*] Yes!

50 I think so!

10 Everybody deserves to learn!

11 Everybody *deserves*. But I don't know if every single museum I visited was like that.

50 Museums are more for grown-ups, generally…

JP We'll get there! We're going to talk about that too!

31 I don't think so… I think it's, like, for all ages.

50 [*Replying to* **31**.] Nobody would bring a *baby*! He can't just be there going «naaaaah» [*imitating a baby crying*] …

[*Laughter.*]

11 Everybody has the right [*to visit museums*], yes, but even if they try to lower [*the cost of entrance*] there are people with no money at all… and they won't be able to go. So I wouldn't say absolutely everyone…

31 [*Completing.*] Has access to the museum.

11 Yeah.

31 We're so smart!

20 Everybody here is smart!

JP Can I move on to the next question? Do you think you like visiting museums more now that you're Little Naturalists?

All [*In unison.*] Yes!

30 It's more fun! We learned more. It seems like the world…

10 It seems like you understand things better!

30 Exactly! Before we were like «What is this?!»…

11 Like, I used to think that one of the marine mammals was an earthworm's ghost!

[*Laughter.*]

20 Now that you know more, it's more fun to learn.

10 And you learn more!

20 Right!

[*Voice overlap.*]

11 Like: when we're four [*years old.*], we go to a museum and we're like: «Huh?!?!» Like: «I don't understand a thing!» Now you're like: «Now I get it!».

[*Laughter.*]

20 Because you understand some things, it's more fun.

11 Then when we become adults, we already know that, we've seen it lots of times!

31 But it's also fun to show your kids!

11 Yes, and not just the children, but the grandparents, also!
[*Voice overlap.*]

31 And to great-grandparents and great-great-grandparents!

JP Do you feel like you've learned something from these activities?

All [*In unison.*] Yes!

JP A lot?

All [*In unison.*] A lot!

10 Yes!

20 It seems like the world is new!

31 It would be wonderful if school science classes were like this!

50 Yes. We would need a garden!

31 [*Laughing.*] It’s true.

[*Voice overlap.*]

30 Because learning science just sitting down isn’t much fun.

31 The only super interesting thing… I mean, it was not super… but the only really interesting thing I did in science classes was observe real rocks and minerals.

11 But we did that here, too! [*Pause.*] But here it's cooler because we had more activities. At school, we just looked at the rocks and didn't write anything! We just passed them around, looked at them, saw the structure...

JP Okay. Can I ask you the next question? What did you learn?

11 A lot!

JP This might be a bit harder. Forget it, let’s move on!

11 But don’t you have to do a test to do for college?

JP I can cut this question out. [*Moving on.*] Of the topics we’ve covered here, which are your favorites? Maybe we should go one by one, okay?

[**JP** *turns to* **51**.]

51 There’s a lot of them…

31 [*Teasing* **51**.] It's the first time I hear him/her talking!

10 I enjoyed the minerals.

20 Everything!

30 Minerals and rocks, and also one we haven't done yet, but I know a few things about, which are invertebrates.

50 Everything!

20 [*Teasing* **50**.] Copycat!

11 I enjoyed… fishes, mammals, minerals…

20 Everything, basically…

[*Laughter.*]

11 Everything, basically.

JP [*Taking notes.*] Everything…

[*Laughter.*]

30 There's no way to choose! Everything is just so fun!

11 But if I had to choose one, I would say… none!

JP None…?

11 None… because I liked them all!

[*Laughter.*]

31 If I had to choose only two subjects, I'd choose fish and mammals! But I would choose everything if I could!

JP What did you enjoy the most, in terms of activities?

10 Today!

31 Today!

50 Yes!

[*Voice overlap.*]

10 To go out and see things…

50 To explore!

20 Putting on the wellies, to explore and go out…

11 I really enjoyed having the little magnifying glasses to look at mosses. It must be the first time I used a real magnifying glass!

20 And the microscope, to actually study!

11 I generally use magnifying glasses to play.

JP And what did you dislike? Or enjoyed less?

11 Nothing!

All [*In unison.*] Nothing…

JP Wasn't there anything you disliked?

31 Seeing 10 and 20 pluck out the fish's eye!

50 I enjoyed that!

[*Laughter. Voice overlap.*]

31 I enjoyed the fish session but, like, that was disgusting…

50 Yeah, I also plucked out the fish's eye!

[*Voice overlap.*]

20 I don't know if you noticed but… we saw all the organs!

50 Yeah, I ripped the fish's heart out…

[*Voice overlap.*]

JP Don't all talk over each other, otherwise it'll be a bit difficult to record... But go ahead!

20 It was weird, but it was kind of…

31 It was gross!

10 It was cool!

11 At least we learned something!

20 And then it smelled bad.

JP So you all agree that the fish-dissecting lesson was the most…

10 [*Interrupting.*] More or less.

50 Let's say, the most disgusting!

[*Voice overlap.*]

20 I like to touch real things…

JP [*Taking notes.*] To touch real things…

11 The lesson I liked the least was the one where we studied the instruments.

50 The scientific instruments.

31 I'd forgotten that session.

20 I don't remember it either…

[*Voice overlap.*]

31 I'd take back the fishes and put that one…

30 It was the most boring.

31 I didn't like that one very much…

50 Yeah, I also found it boring.

31 It was a bit.

JP You didn't find it fun?

All [*In unison.*] Yeah…

31 I enjoyed building the compass afterwards but, like…

[*Voice overlap.*]

31 But then it was kind of boring.

20 But the compass was cool… We all working there…

[*Laughter.*]

10 The ending was cooler.

JP [*To* **51**.] You have nothing to say, **51**?

20 He/she never speaks!

31 It was the first time I heard this kid speaking!

31 I only heard **51** speaking once!

20 But that doesn’t matter for the interview!

JP Was there anything you didn't like? Or that you liked less? Did you like everything equally?

51 [*Quietly.*] Yes…

JP Ok. So, what would you like to do here that you haven't already done?

11 [*Thinking.*] Uh…

31 Sheesh! A lot of stuff!

11 Studying the reptiles!

JP I think you *are* still going to study the reptiles!

20 We are!

11 But we haven’t done it yet!

31 What I don’t want to do is to study the spiders!

10 I would like to do a more aquatic activity!

JP More aquatic?

11 [*Teasing* **10**.] Like what? Going inside a swimming pool…

JP [*Joking.*] Put on a wetsuit and jump into the water?

10 No!

[*Laughter.*]

JP [*Recapping.*] But what would you like to do that you haven't done yet?

31 I've already said what I wouldn't like. But what I would like… Uh, I would like to… Clearly… Obviously… I don't know.

[*Laughter.*]

31 It would be very hard to tell.

JP Would you like to move on?

20 Because we haven't done it yet, we can't imagine what it will be like, because we don't see the future like a fortune-teller.

31 I think I'd like to have just one animal topic: nocturnal animals.

JP That would be nice! I think it's here in the Biodiversity Gallery that they do an initiative from time to time: they take people for a walk around the garden to see the nocturnal animals.

11 At night?!

JP At night! But maybe I'm confusing things…

11 That would be cool!

JP But maybe I'm confusing things. I don't know whether it is here or in the City Park... Anyway...

50 We can all go on an activity at night! Instead of being in the afternoon, it would be at night!

30 There was already an activity about nocturnal butterflies!

JP It's a matter of organizing things! [Moving on.] Would you take part in this project again if you could?

All [*In unison.*] Yes!

11 I would participate until I was your age!

[*Laughter.*]

11 [*To* **JP**.] How old are you?

JP I'm 23.

11 What?! I thought you were 15 or something!

50 [*To* **11**.] Really?! *Fifteen*?!

JP Thank you! [*Joking.*] I am well preserved, don't you think?

[*Laughter.*]

JP But so, you all agree that you would take part in this activity again, right?

All [*In unison.*] Yes!

? Absolutely!

JP And would you come back to visit the Central Pole or the Gallery here, even without taking part in this activity?

All [*In unison.*] Yes!

JP You know what the Central Pole is, right?

50 [*Joking.*] No…

[**JP** *was in doubt. Everybody started laughing.*]

30 That's where the Ferreira da Silva Laboratory is, isn't it?

JP That's where the laboratory is, right!

31 Oh. I've never been there but I think I know what it is…

JP Okay, right. But if you could, you'd all come back here to the Gallery, right?

All [*In unison.*] Yes!

JP Right! Now, this has to do with something that was said earlier: do you think museums are appropriate for your age or do you think they're for older people?

50 It depends!

10 It depends on the subject of the museum!

50 Sometimes it seems to be more for adults, other times...

20 Like, if it's the human body, like, the heart and stuff, I think it's more for adults…

10 The veins!

[*Someone expressed disgust: «Blergh!»*]

11 I think it's for all ages for one reason and one reason only: because museums don't have just one thing, they have many things!

50 Exactly! But like…

[*Discussion. Voice overlap.*]

31 You can make a subject out of a thousand things…

11 A lot of things about just one subject, but they all seem different, except that it's... just plants, just plants...

31 If it's, like, a museum with just one subject, like dinosaurs, then I admit I'd love it. I'll give you that! But any other subject, I don't think so... Only dinosaurs...

JP Right!

10 It's over?

JP But do you think that the way museums are organized* takes into account people your age or do they only think about adults**?

50 Yes!*

11 Yes!*

50 No!**

11 No!** They have…

20 No!**

10 No!**

11 I say: all ages!

50 Yes!

20 Me too!

10 And I!

20 Look, **10** agrees with me!

JP Well, I think the session is over! Thank you!
