# Supplementary material for: Children’s perceptions of natural history museums: Insights from the “Young Naturalists” program in Portugal
Source: PLoS One. 2026 Sep 18;21(9):e0357466. doi: 10.1371/journal.pone.0357466 (PMC13588495; doi:10.1371/journal.pone.0357466)
Supplement: Supplementary file 3 [file pone.0357466.s003.pdf]

FOCUS GROUP C

Players: JP, MF, 13, 14, 15, 34, 35 and 44

JP This is for an assignment I have to do for college. Essentially, I need to analyze the ideas that children and youngsters have about museums. I ask you not to talk over each other. I'm going to put a few questions to you, but as well as answering me, you can also talk to each other so that there's a bit of interaction. The first question I'm going to ask you is: what is a museum? If someone asked you to explain what a museum is, what would you say?

[**JP** *addresses each person.*]

13 I think a museum is a place where you keep really old and valuable stuff.

[**34** *laughs at* **13***'s informality.*]

34 [*Laughing.*] So, I think a museum is somewhere that basically safekeeps our history, our evolution.

15 I think a museum is a place that keeps valuable things that are old, which people can see and admire.

JP [*Taking notes.*] Nice!

35 A museum can be a building, but there are cases where it isn't. It usually has a theme and a collection of things related to that theme and a large part of that collection, whatever the theme is, is generally open to the public to help explain it to the public and expand the public's knowledge of it.

JP Please note: it's not compulsory for you all to tell me what a museum is in detail. It's just so I can have an idea... If you don't want to say any more, and if you think that your peers have already said everything there is to say...

44 I think the most important thing for a museum is to help people not forget what happened and how we began existing and why...

JP [*To* **14**.] Do you want to answer the question, **14**?

[**14** *cringes.*]

JP We'll move on... Now: this question probably answers the previous question a little: what do you think is the point of a museum? What do you think a museum's functions are?

13 To store very old stuff safely, so they don't get stolen.

[**34** *laughs at* **13**'s *vocabulary.*]

JP [*Reiterating.*] So they don't get stolen?

13 Like, valuable minerals!

JP Nice! Good point!

34 Yeah... It's basically what we answered earlier.

JP Yeah, maybe it's better to skip this one...

34 It's about saving history and stuff...

JP Do you like visiting museums?

All [*Unison, quietly.*] Yes.

JP Everyone says yes, right?

34 Yes...

JP And do you think you learn something when you visit museums?

All [*Unison.*] Yes.

34 Of course!

13 That's why we have a guide that explains everything to us!

[*Laughter.*]

JP Nice... And how do you think the objects in museums end up there? The stuffed animals, the artifacts...

13 They're found! By archaeologists...

JP [*Reiterating, taking notes.*] By archaeologists...

34 And not just archaeologists!

35 [*Reiterating.*] And not just archaeologists...

34 By geologists as well!

34 Or experts in the field...

35 There can be a lot of work done by a lot of people that maybe wasn't even for museums originally. It was more for private galleries and so on, but nowadays it's for museums. It's because of the work of all these people that museums exist.

JP You have a good notion of the history of museums! Very good!

44 A lot of the things I know that went to museums came from people who used to embalm animals, find things, and study them. The only way to study an animal [*brought from another country*] was to embalm it. And as for plants, you had to grow them. Many things were passed on to warehouses or museums.

JP Exactly! Since there were no photographs, or anything like that, it was complicated... [*Silence.*] Nothing? Well... [*Continuing.*] What precautions do you think we need to take?

[**13** *puts his/her finger in the air.*]

JP Go on!

13 Like, embalming the animal very carefully...

JP Sorry! What I meant now was: what care do *visitors* have to take in the museum?

13 Oh, the *visitors*?

JP Yes, the visitors.

13 Not touching without the guide's guidance... Where it's written "Don't touch" you just can't touch! It's forbidden. If you do, you have to be very careful not to damage it!

JP That's right!

34 If you have to touch it, you've got to use protection, something appropriate, so you don't damage the object.

15 You've got to be very careful, so if you have to touch something, you use protection.

35 Yes! Museums must protect their pieces and visitors must respect that: they must try to see in a non-invasive way and... well, *to see with their eyes!*

44 You shouldn't take photographs without permission. It's preferable never to use flash, even if you can. You can always ruin it. And maybe not touching. There are people who knock on the glass and that can ruin things.

JP Well done! Nothing to say, 14?

[**14** *shudders and shakes his/her head.*]

JP Okay... And why do you think it's important to preserve and protect the objects in museums?

34 So people can know about things from the past and admire certain things.

35 We need increasingly more knowledge. For example, history museums are very beneficial for the population because most people don't have a clue about history and that can create some misunderstandings and controversies. We need the general public to learn more about various subjects. For example, even for things that we don't give any importance to, we need to show the process and the history of these things, and the thousands of people who sacrificed their lives for it...

JP Nice!

[**JP** *turns to* **44**.]

44 Uh... What was the question?

JP Why do you think it's important to preserve objects in museums?

44 It's always important, mainly because we need to know what happened. And in the future, there may be things for which we will need the knowledge that came from the past and if we don't have that concern with preservation, we may not be able to understand those things in the future.

JP Nice! Does anyone else want to add anything?

34 I think it's just that everyone has the right to learn and know about evolution, the processes of nature...

JP Nice!

13 Museums have video surveillance too!

34 Yes, but see, if someone does something, video surveillance won't help you recover what's been damaged.

13 Yes, but you find out who did it!

34 I know, but...

[*Discussion, voice overlap, laughter.*]

35 The aim isn't to find out who did it, it's to make sure they don't do it!

34 So that the objects don’t get damaged.

15 Exactly!

JP Well, can I move on to the next question?

All [*Unison.*] Yes!

JP This question is a bit weird: do you think museums are available to everyone equally or do you think our society is a bit unfair because... because museums are more for rich people?

34 [*Thinking, not sure what to say.*] Uh...

JP That's what I'm saying: this question is probably a bit crazy...

13 I don't agree!

35 Well, I would say that most museums used to be for rich people. There was nothing [*for the public*]. And there has been a gradual effort to make information available at an affordable price or even free of charge - an effort that remains to be seen, but today, thank God, most museums are already for the public.

34 Exactly, now there's even like a family ticket or a children's ticket. So, it becomes more accessible!

JP Yes, we'll talk about the issue of children later. But it’s good that you brought it up! [*To* **13**.] You wanted to say something, didn't you, **13**?

13 Of course! I think it should be available to everyone, no matter who they are! Poor, rich...

JP Right! Because sometimes it's not even about rights, because we all have the rights, it's about money.

35 It's also about *power*.

JP Money, purchasing power...

35 The state and things like that...

15 When I came here recently, I wanted to buy something, a toy, but it was too expensive. My parents didn't let me.

44 I think it's important that it's free or cheap because in the past only rich people could study and know certain things. And we all need to have the same amount of knowledge so humanity can evolve. In the old days, it was actually kind of stupid because a lot of rich people didn't even care about these things.

JP Exactly, is there anything else you'd like to say?

35 I don't think so...

15 No.

JP Do you think you like visiting museums more now that you're Young Naturalists? Or is it the same?

13 I like it more now!

JP You like it more?

13 Yes!

34 I think I've come to like it better too. In a way, it gives us another point of view.

15 I feel we have more knowledge!

34 Exactly! Then we become more interested...

35 I already enjoyed visiting museums before, but now being almost involved here gives you another point of view, completely different. You can also appreciate the work that went into making this museum and all the people who have contributed...

44 Now we have a bit more knowledge about different areas and now we can try to go to different museums. We can understand things a bit better now.

JP [*To* **14**.] Nothing to say?

[**14** *shudders.*]

JP Okay. [*Moving on.*] Do you feel you've learned anything from these activities?

All [*Unison.*] Yes!

JP A lot? a little?

15 A lot!

34 Yes!

35 Yes. What I'd already learnt I've now learned from another point of view because learning at school and learning from people who understand the subject and are interested in it is completely different! At school it’s all about copying, memorizing, and forgetting...

[**15** *laughs.*]

JP Yeah! [*To the group.*] You want to say anything else about this?

15 Uh... No.

JP Now: which of the areas covered do you think are your favorites? Maybe we should go one by one again.

13 For me it was the plants.

JP Plants? Good!

34 I also enjoyed the part about plants, and that session where we dissected a fish.

15 [*Laughing.*] I think I liked dissecting a fish, too.

13 **14** cut the fish in two!

15 [*Continuing.*] And... I think we've already had the insects session. So, I'll pick that one too.

JP Everyone loves dissecting the fish!

[*Laughter.*]

35 Honestly, everything. I don't think I can choose one. They've all been good so far.

44 I preferred the fish activity and the paleontology and geology activity.

JP [*Reiterating, taking notes.*] Fish and paleontology and geology, right?

44 Yes.

JP Nice!

14 I think I liked the plants and mammals the most.

JP Good! Now: what didn't you enjoy? Or enjoyed less?

35 That's the hardest question so far!

[*Laughter.*]

34 I don't think I have anything to say about that...

JP Don't you have anything to say? Did you enjoy everything equally? Wasn’t there anything that you…

34 [*Interrupting.*] Yes, because everything was always dynamic.

JP Mind you: you may have liked everything, but there may have been something that wasn’t quite up your alley.

35 The wellies were a bit uncomfortable!

[*Laughter.*]

34 Yes, the wellies!

15 The wellies, that's right!

35 Apart from that, the idea is good, and the execution is perfect!

15 Everything except the wellies...

JP And what would you like to do here that you haven't done yet?

13 The human body!

35 [*Laughs.*] We would need to dissect a human for that!

[*Laughter.*]

34 [*Laughing.*] How horrible! No!

JP [*Joking.*] Suggest that to **MF**...

[**MF** *laughs.*]

13 We could get a fake [*a dummy*].

44 Studying reptiles!

JP But do you have any other ideas that you'd like to suggest? Feel free!

35 We’ve already done all the ideas I've thought of.

13 I was going to suggest one outside of here...

JP Go on!

13 Engineering!

JP Engineering? There was a session about scientific instruments, I think...

15 There was. I think it was the first one. I was there.

13 It was the first! It was the first!

35 Not the first, the second. The first one was the introduction.

JP There was that activity in the Ferreira da Silva Laboratory where you drew the instruments in the lab, but I think there was another one. Maybe I’m confused! [*Continuing.*] Would you take part in this project again if you could?

All [*Unison.*] Yes!

35 100%!

JP And would you visit the Central Pole or the Biodiversity Gallery again?

All [*Unison.*] Yes!

35 And we'd visit them under a different light!

JP Now, this has to do with something your friend has already said: do you think museums are appropriate for your age? Or are they more for adults...?*

13 [*Answering straight away.*] No!*

34 No!*

15 No!*

JP Some of you are practically adults!

34 There are museums that are more dynamic and there are others that are... well, not *boring*, but...

15 Some have things that can sometimes scare people.

JP Scare people? Like what? Can you name something?

15 Uh... I think there was one time when my cousin, when he was younger, went on a field trip to a museum and he fainted... [*Laughing shyly.*] It was something about the human body he had seen.

13 I also went to an exhibition of the human body once and almost went "Blergh!" [*mimics vomiting*]

35 That also depends on the person's knowledge and what they do. It doesn't have so much to do with age, it can have more to do with maturity, with tastes, for example, I can go and see a museum of... [*thinking*] African colonial history and I might find it interesting and 90% of people might find it uninteresting.

13 Exactly!

34 It also depends on people's tastes, not just their maturity. And interests.

JP It also depends a bit on each person's sensitivity, don't you think? You talked about the human body, there may be people who find it a little more uncomfortable. Or even history! When you go to a museum to study the bad things that have happened in history… I think they've put on a Holocaust exhibition nearby, for example... So, I guess that's it! Thanks, guys!
